# In vivo characterization of carbon dots–bone interactions: toward the development of bone-specific nanocarriers for drug delivery

**DOI:** 10.1080/10717544.2021.1938753

**Published:** 2021-06-26

**Authors:** Rachel DuMez, Esmail H. Miyanji, Lesly Corado-Santiago, Bryle Barrameda, Yiqun Zhou, Sajini D. Hettiarachchi, Roger M. Leblanc, Isaac Skromne

**Affiliations:** a Department Biology, University of Richmond, Richmond, VA, USA; b Department Chemistry, University of Miami, Coral Gables, FL, USA

**Keywords:** Carbon nanodots, C-dots, nanocarriers, theragnostic, bone disease, osteoporosis, zebrafish, appositional growth, bone, skeleton

## Abstract

Current treatments for osteoporosis and other bone degenerative diseases predominately rely on preventing further bone erosion rather than restoring bone mass, as the latter treatments can unintentionally trigger cancer development by undiscriminatingly promoting cell proliferation. One approach to circumvent this problem is through the development of novel chemical carriers to deliver drug agents specifically to bones. We have recently shown that carbon nanodots (C-dots) synthesized from carbon nanopowder can bind with high affinity and specificity to developing bones in the larval zebrafish. Larval bones, however, are physiologically different from adult bones in their growth, repair, and regeneration properties. Here we report that C-dots can bind to adult zebrafish bones and that this binding is highly specific to areas of appositional growth. C-dots deposition occurred within 30 minutes after delivery and was highly selective, with bones undergoing regeneration and repair showing higher levels of C-dots deposition than bones undergoing normal homeostatic turnover. Importantly, C-dots deposition did not interfere with bone regeneration or the animal’s health. Together, our results establish C-dots as a potential novel vehicle for the targeted delivery of drugs to treat adult bone disease.

## Introduction

Bone diseases such as osteoporosis affect over 54 million individuals annually in the US alone (Wright et al., 2014), imposing a heavy burden to society that could be mitigated with improved diagnostic techniques and expanded treatment options. For osteoporosis, early diagnosis is key for the most successful treatment prognosis, as current treatments predominantly rely on preventing further bone erosion and not in restoring bone mass, as drugs that promote bone growth can lead to cell proliferation in other tissues and increase a patient’s risk to cancer (Marie & Kassem, 2011). Bone disease diagnostics rely on X-ray imaging methods and MRI or CT scans, with novel, higher sensitivity, fluorescent-based technologies currently under development (Chen et al., 2014; Gruneboom et al., 2019; Jung et al., 2019; Yan et al., 2019). The development of more sensitive diagnostic tools for early bone-loss detection and novel treatment methods for stimulating bone growth without affecting other tissues will significantly ameliorate the societal and personal cost of bone diseases.

Carbon-based nanoparticles or carbon dots (C-dots) have emerged as novel therapeutic and diagnostic biomaterials due to their unique, tunable physicochemical properties (Zhou, Mintz, et al., 2019; Dan et al., 2020). Common properties shared by all C-dots include small size (<10 nm), high carbon content, robust photostability, and bright fluorescence (Mintz et al., 2019; Zhou, Mintz, et al., 2019). While several physicochemical factors contribute to these properties, the most influential are core configuration and surface functional groups (Mintz et al., 2019; Yan et al., 2019; Zhou, Zahran, et al., 2019). Importantly, the functional groups at the surface can be modified during their synthesis to fine-tune the C-dots’ photoluminescent properties (Zhou, Mintz, et al., 2019; Dan et al., 2020), or to adapt C-dots to function as pharmaceutical nanocarriers (Zheng et al., 2014; Zhou, Liyanage, et al., 2019). For example, the surface of C-dots can be passivated with polyethylene glycol (Sun et al., 2006; Peng et al., 2020), and then conjugated covalently or noncovalently with diverse therapeutic agents drugs (Thakur et al., 2014; Iannazzo et al., 2017; Hettiarachchi et al., 2019; Liyanage et al., 2020). These modifications, however, can have unexpected consequences for both the C-dot vehicle and the drug cargo, including affecting the solubility, therapeutic efficacy, and systemic clearance of the drug (Misra et al., 2015; Li, Amat, et al., 2016; Li et al., 2020). To fully exploit C-dots’ theragnostic potential, it is imperative to first characterize unmodified C-dots.

We recently described that C-dots synthesized from carbon nanopowder have a high affinity and specificity for developing zebrafish bones (Li, Skromne, et al., 2016; Peng et al., 2017). When carbon nanopowder**-**derived C-dots were injected into 5-day old zebrafish larvae, their deposition was observed in opercular (intramembranous) and vertebrae (endochondral) bones, but not in non-skeletal tissues (Li, Skromne, et al., 2016; Peng et al., 2017). C-dots deposition on bones was dependent on mineralization, as manipulations promoting or interfering with bone mineralization increased or decreased C-dot deposition respectively (Li, Skromne, et al., 2016). Of note, C-dots binding to bones was only observed when the C-dots were synthesized from carbon nanopowder through oxidation in hydrothermal conditions, and not when synthesized from citric acid or EDA using solvothermal methods (Peng et al., 2017). This difference in binding may be due to the presence of different chemical groups at the surface of the C-dots: while nanopowder-derived C-dots are rich in negatively charged carboxyl groups that can interact with positive calcium ions in the bone’s matrix, the surface of citric acid- and EDA-derived C-dots is rich in positive amino groups (Peng et al., 2017). These results suggest that the unique physicochemical properties of carbon nanopowder C-dots are critical for their binding to the larval mineralized bone.

Zebrafish provides a robust, *in vivo* model to test C-dots’ interaction with biological tissues, as their transparency allows direct observation of C-dots’ photoluminescence. Importantly, bone formation and maintenance in zebrafish are remarkably similar to that of other vertebrates (Witten et al., 2017; Busse et al., 2020; Tonelli et al., 2020). During embryogenesis in fish, birds, and mammals, bones develop either through the direct aggregation of bone-forming cells (intramembranous ossification; e.g. cranial bones) or through the deposition of a mineral matrix on a collagen scaffold (endochondral ossification; e.g. long bones) (Salhotra et al., 2020). Once formed, adult bones undergo homeostatic turnover and can continue to increase in diameter through the process of appositional growth, whereby new mineralized tissue is added to the bone’s surface (Rauch, 2005; Suniaga et al., 2018; Salhotra et al., 2020). These appositional growth and remodeling processes are carried out in zebrafish by similar skeletal cells and ossification mechanisms that have been observed in mammals (Busse et al., 2020; Tonelli et al., 2020). In addition, zebrafish can rapidly regenerate caudal fin tissue, including bony fin-rays, in less than 6 days (Busse et al., 2020; Tonelli et al., 2020). This remarkable regenerative capacity, together with transparency and thinness, makes the caudal fin of the adult zebrafish an excellent model to study bone homeostasis, repair, and regeneration.

Here, we investigated the interactions between carbon nanopowder C-dots and skeletal tissues of adult zebrafish, reporting that C-dots deposit on adult bones undergoing homeostatic turnover, appositional growth, and regeneration. This is an important first step toward developing C-dots as theragnostic agents for the treatment of adult bone diseases. We report that, irrespectively of delivery method, C-dots bind with high affinity and specificity to mineralized adult bones, particularly those undergoing rapid growth. C-dot deposition occurred within the first 30 minutes after injection and was found to co-localize to Alizarin Red Complexone (ARC), a well-known histological marker of areas of bone growth (Hoyte, 1960). Importantly, C-dots deposition to bones did not interfere with normal bone physiological processes and growth, or affect animal’s viability. Taken together, our results highlight the vast potential of photoluminescent C-dots as a tool for the diagnosis and treatment of adult bone diseases.

## Materials and methods

### Carbon dot synthesis and analysis

Carbon nanodots (C-dots) were synthesized from carbon nanopowder and purified using our previously reported procedure (Li et al., 2015; Li, Skromne, et al., 2016). To obtain C-dots in powder form, the C-dot solution was lyophilized in a Rotovap evaporator at 60 °C. The morphology and photoluminescent properties of as-prepared C-dots were confirmed using transmitted electron and epi-fluorescence microscopy, as previously described (Li et al., 2015; Li, Skromne, et al., 2016).

### Zebrafish care, tail amputation, injection, and bone staining

Wild type (TAB-5; (LaFave et al., 2014)) and Casper (mpv17^a9^; mitfa^w2^; (White et al., 2008)) zebrafish were obtained from the Zebrafish International Resource Center (Eugene, OR) and maintained at the University of Richmond animal facility following standard husbandry protocols (Westerfield, 1995). All protocols and procedures were reviewed and approved by the Institutional Animal Care and Use Committee. All experiments were performed on animals 4–6 months of age. For amputations, fish were fully anesthetized in 0.2 mg/ml Tricaine (pH 7.0) until unresponsive to touch, placed on an inverted glass Petri dish covered with Parafilm, and a sterile blade was used to cut 40 mm away from the distal tip of the caudal fin. For injection, fish were anesthetized and weighed to standardize the mass of C-dots injected per body weight (µg/g). Then, fish were positioned on a wet sponge under the microscope and injected intraperitoneally or intravascularly with C-dots using a 36G Nanofill microsyringe attached to an electronically controlled micropump (UMP3 UltraMicroPump, WPI), as previously described (Pugach et al., 2009; Kinkel et al., 2010). After manipulations, fish were allowed to recover for 30 min and returned to the animal facility where they received standard care for the duration of the experiment.

Live staining of mineralized structures was done using Alizarin Complexone (ALC; Sigma-Aldrich, A-3882). First, fish were sedated in Tricaine solution in fish facility water (0.1 mg/ml; pH 7.0). Then, fish were stained in a solution of ALC (10 mg/ml) and Tricaine (0.01 mg/ml; pH 7.0), for 30 min. To remove excess dye following exposure, fish were quickly rinsed in three sequential washes of fresh facility water and allowed to recover from sedation for 60 min before collecting the caudal fins for histology. Following fin collection, fish were allowed to recover as above.

### Histology

For histological sectioning, fish were anesthetized and the regenerated fin was amputated at a site proximal to the original cut. For unmanipulated controls, the cut was done 40 mm away from the distal tip of the caudal fin. Immediately after caudal fin collection, the fish was placed in a recovery tank and the fin was splayed out by floating it on a large drop of 4% paraformaldehyde in 1x PBS inside a Petri dish. After a few min, once the fin was submerged in the droplet, the Petri dish was closed and the fin was fixed for 4 h. The fixative was then removed and replaced with a 10% sucrose solution. The Petri dish was sealed and stored at 4 °C overnight. Samples were processed within 5 days. Fins were embedded in Clear Frozen Section Compound (VWR), frozen, and sectioned at 5 microns in a Leica CM1520 cryostat. Sections were collected on positively charged slides (Globe Scientific), allowed to dry for 30 min, and covered with mounting media (30% glycerol in 1x PBS, 5 mg/ml n-Propyl gallate, 2.5 µg/ml DAPI). Slides were kept in the dark at 4 °C and sections were imaged within one week.

### Imaging and analysis

For imaging, zebrafish were anesthetized and placed on a glass Petri dish with the caudal tail splayed to separate the fin rays. Whole fin images were acquired under brightfield and epi-fluorescent light using a Zeiss Discovery V.20 dissecting microscope and a Zeiss Axiocam MRc camera. Filter sets for Fluorescein (green; 488/525 nm) and Texas Red (red; 596/615 nm) were used to detect C-dots and ALC, respectively. For consistency across experiments and biological replicates, all fluorescent images were taken using time exposures of 1 and 2.5 s. Images were processed using Zeiss AxioVision SE64 v4.9.1 and fluorescence quantification was done using ImageJ v1.52. Fin sections were imaged using an Olympus Fluoview mounted on a fully automated Olympus IX83 inverted confocal microscope, using the company’s imaging software package. Appropriate wavelengths were used to detect C-dots and ALC. Representative images were cropped and assembled into figures using Adobe Photoshop v21.2.

Quantification of C-dots deposition on bones was based on the intrinsic photoluminescent properties of C-dots (Li, Skromne, et al., 2016). Fluorescent images of control and experimental fins were converted to 8-bit grayscale images using ImageJ, and the pixel intensities in areas of interest were obtained. In both control and experimental conditions, intensity values in regenerating areas were subtracted from background levels of non-regenerating regions. Then, intensity values in experimental conditions were normalized relative to control conditions. The integrated intensities for each region were averaged to determine relative fluorescence in arbitrary units for each treatment group. For signal profile analysis, the scale was set to 1 pixel = 1 µm and a 1300 µm straight line was drawn starting 300 µm anterior to the cut site along the 4th ventral-most ray. The signal profile was generated using the Analyze > Plot > Profile function of ImageJ. Signal intensity in regions of interest or along the profile line was recorded and analyzed in Excel. Graphical visualization of data was done in Excel or RStudio. The RStudio code used for fluorescence analysis is available as Supplemental Material on the publisher’s webpage.

## Results and discussion

### C-dots bind to adult bones undergoing homeostatic turnover, repair, and regeneration

**Figure 1.**
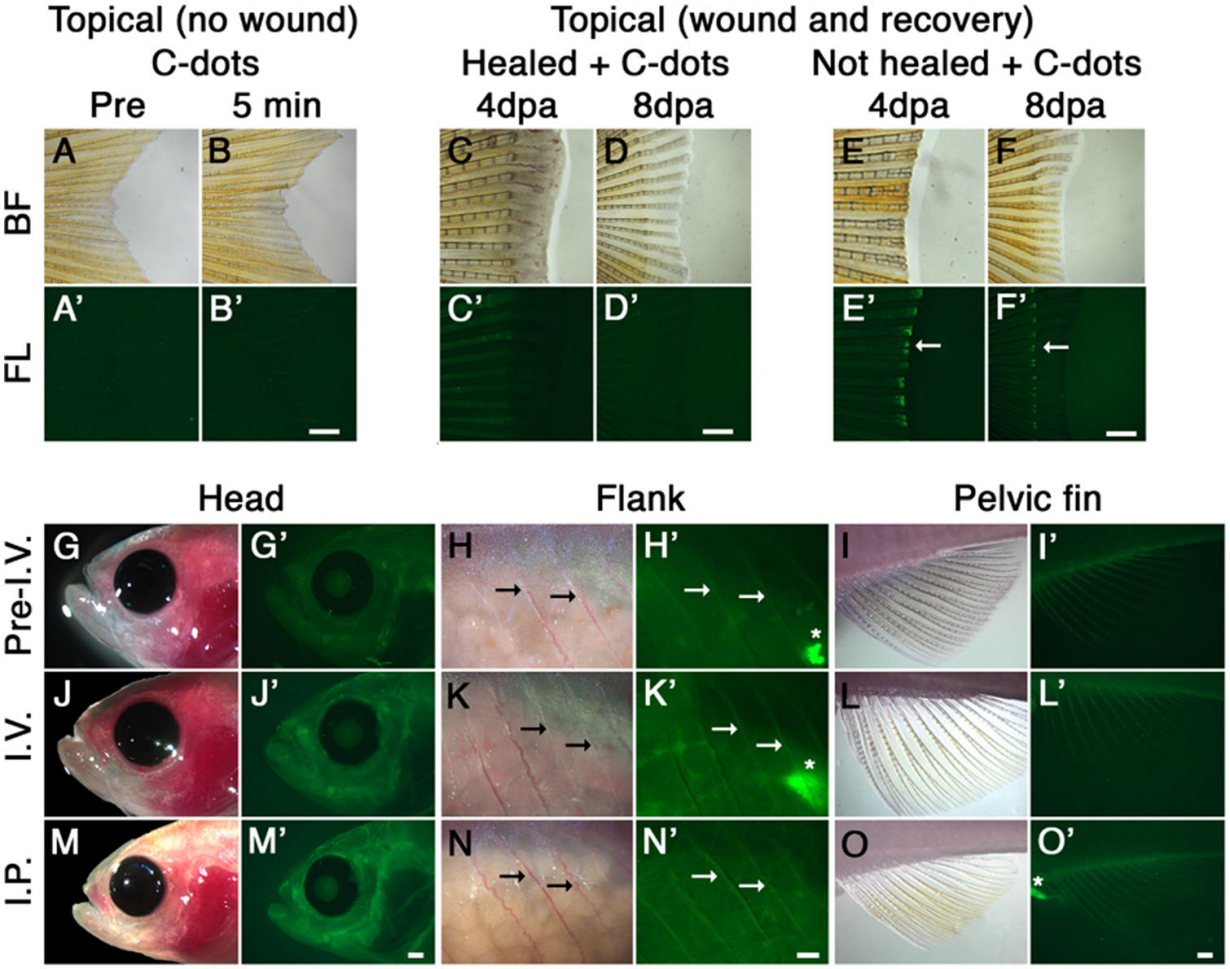
C-dots binding to adult ossified tissue. (A, B) Bright field (BF; A, B) and fluorescent (FL; A’, B’) images of caudal fins before (A, A’) and 5 min after (B, B’) cutaneous exposure of C-dots to skin (2 min; 2 µg/ml). (C, D) Similar to B, cutaneous exposure of C-dots to a healed wound 4 and 8 days post amputation (dpa) does not label local bones. (E, F) Cutaneous exposure of caudal fins to C-dots immediately after amputation resulted in bone labeling at the wound site (E, E’; green fluorescence; white arrow). Four days later, C-dots can be observed in bone tissue at the wound site, but not in new bone tissue (F, F’). (G–I) Images of head, flank and pelvic fin of fish prior to intravascular injection (Pre-I.V.) of C-dots under reflected bright field and epi-fluorescent illumination. Thick tissues naturally display a low level of background fluorescence. (J–L) Intravascular injection (I.V.) of C-dots resulted in fluorescent labeling of ossified tissue 1 h after injection. (M–O) Intraperitoneal injection (I.P.) of C-dots also resulted in fluorescent labeling of bones 1 h after injection. *n* = 6 fish per experimental condition, in two independent experiments. Fish are positioned anterior to the left and dorsal to the top. In flank images, arrows indicate ribs and asterisks nonspecific gut autofluorescence. Day post amputation is indicated as dpa. Scale bars are 500 µm.

To gain insight into the adult theragnostic potential of carbon nanopowder-derived C-dots, we analyzed their toxicity, bone binding dynamics, and photoluminescence in transparent adult zebrafish (Casper strain (White et al., 2008)). We analyzed C-dots’ bone deposition using standard 488/525 nm excitation/emission Fluorescein filter set to capture their intrinsic photoluminescence (excitation peaks between 360–540 nm and emission between 500–600 nm (Li, Amat, et al., 2016)). Three different delivery methods were used to determine the ability of C-dots to bind adult bones, one cutaneous and two through injection (Figure 1). To determine if C-dots can be adsorbed cutaneously, we applied a concentrated solution of C-dots to the caudal fin or the flank of the fish. Neither application resulted in the labeling of local bones (Figure 1(A,A’,B,B’) and data not shown). Similarly, topical application of C-dots to a healed caudal fin wound undergoing regeneration did not label local bones (Figure 1(C,C’,D,D’)). In contrast, C-dots applied to a wound with exposed bones labeled the exposed tips of the bones (Figure 1(E,E’)). Notably, none of the bone tissues that regenerated following C-dots exposure were labeled, indicating that following deposition, C-dot binding became fixed (Figure 1(F,F’)). We next employed two injection delivery methods, intraperitoneal and intravascular (retro-orbital). Delivery of C-dots (20 µg/g fish) through either method effectively labeled intact cranial, ribs, and fin bones (Figure 1(G–O,G’–O’)). This labeling was most apparent in the fish fins because of the tissue’s reduced thickness and isolated position (Figure 1(G–O,G’–O’)). These results indicate that direct wound application and injection methods are effective for delivering C-dots into adult fish, with intravascular injections resulting in the most consistent deposition of C-dots on bones (not shown). Together with our previous results in larvae (Li, Amat, et al., 2016), this suggests that C-dots can bind with high specificity to skeletal elements irrespective of bone age.

**Figure 2.**
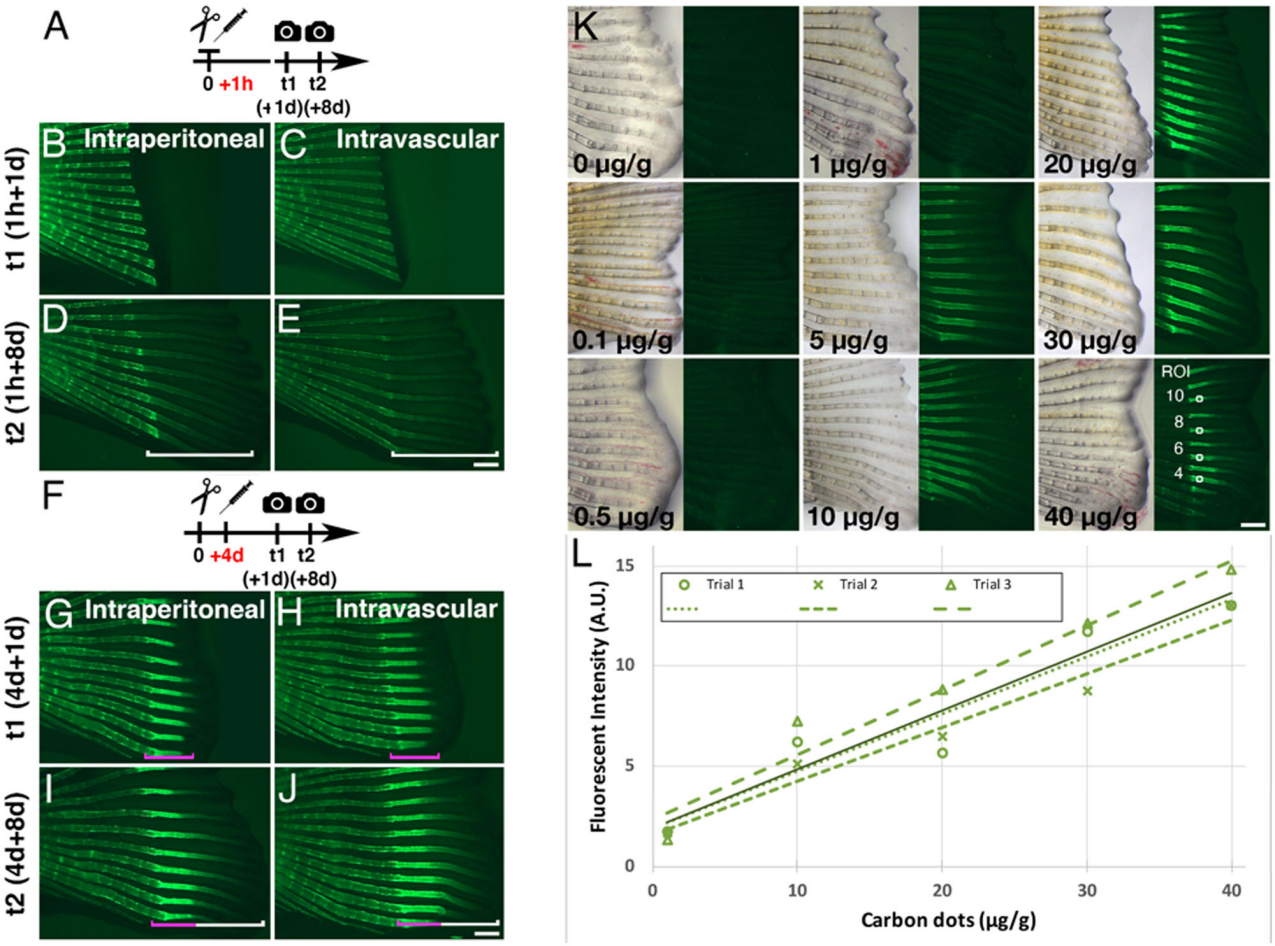
C-dots bind to regenerating bones in a concentration-dependent manner. (A) Schematic representation of regeneration experiment in (B)–(E), were C-dot injection followed caudal fin amputation by 1 h. (B–E) Intraperitoneal (B, D) or intravascular (C, E) injection of C-dots before regeneration begins does not label regenerating bone. Images were taken 1 (B, C) and 8 (D, E) days after C-dot injection. (F) Schematic representation of regeneration experiment in (G)–(J), were C-dot injection followed caudal fin amputation by 4 days. (G–J) Intraperitoneal (G, I) or intravascular (H, J) injection of C-dots after caudal fin regeneration has initiated label the bone being regenerated at the time of injection (magenta bracket), but not bone that regenerated after the injection (white bracket). Images were taken 1 (B, C) and 8 (D, E) days after C-dot injection. *n* = 6 fish per experimental condition, in two independent experiments. For all experiments, times 1 and 2 images (t1, t2) are of the same fish. (K) C-dots deposition on regenerated bones correlates with concentration of C-dots injected. Timeline of experiment as in F, except that images were collected 4 days post injection. C-dots’ concentration was normalized to fish weight (µg/g; 0 is saline-injected control). Representative regions of interest (ROI) used for fluorescence intensity quantification are indicated with squares for the 40 µg/g injected fish fin. (L) Mean fluorescent intensity of bone-bound C-dots at ROIs as a function of C-dots dosage. Values were normalized to background and saline-injected controls and reported in arbitrary units (A.U.). Dashed lines represent trendlines for three independent experiments (*R*^2^ > 0.91; df = 3, *p* < 0.006), with the solid line representing the mean average trendline (*R*^2^ > 0.96; df = 3, *p* < 0.002). Scale bars are 500 µm.

To explore the binding of C-dots to wounded and regenerating adult bones, we administered C-dots to adult zebrafish that had undergone amputation of the caudal fin and were at different stages of regeneration. This approach allowed us to examine the binding of C-dots to fin ray bones undergoing normal homeostatic turnover as well as regenerative growth. When C-dots were delivered prior to the initiation of fin bone regeneration at 1 day post-amputation (dpa; Figure 2(A)), regenerated bones examined at 8 dpa were not labeled (Figure 2(B–E)). However, when C-dots were delivered during active fin bone formation (e.g. 4 dpa; Figure 2(F)), regenerated bones were strongly photoluminescent (Figure 2(G–J)). The observation that C-dots delivery at 4 but not 1 dpa labeled regenerating bones at 8 dpa suggests that C-dots were quickly cleared from circulation. Attempts to determine if C-dots were metabolized or excreted were unsuccessful, as C-dot concentrations were below the detection limit of our assays (ELISA and TEM; data not shown). Therefore, at this moment, we cannot rule out the possibility that C-dots were removed from the circulatory system through mechanisms other than bone deposition. Nonetheless, once in bones, C-dots label could be detected for at least eight weeks (data not shown), suggesting that binding to adult bones may be permanent.

### C-dots deposition on regenerating bone is dose-dependent, swift, and homogeneous

The photoluminescence intensity of C-dots bound to regenerating bones was qualitatively more intense and homogeneous than the binding to non-regenerating bones, providing an assay to study C-dots binding to ossifying tissue. To begin testing the magnitude of C-dot binding to regenerating bone as a function of C-dots dosage, we repeated the amputation experiment followed by the intravascular administration of increasing amounts of C-dots (Figure 2(F)). The amount of C-dots in solution delivered to each fish was normalized to the weight of the fish (µg/g). The lowest amount at which we were able to detect C-dots binding to regenerating bones was 5 µg/g (Figure 2(K)). The photoluminescence intensity increased until 40 µg/g, the highest dose tested (Figure 2(K)). Higher concentrations were not tested because concentrations above 40 µg/g clogged the microneedles used for injections and the use of larger needles damaged the fish’s vasculature at the site of injection. To quantify C-dots’ binding, we measured the photoluminescence intensity of a 100 × 100 µm square area located 100 µm away from the amputation site in regenerating fin rays 4, 6, 8, and 10 (ventral to dorsal position). These values were averaged and normalized to the intrinsic background of corresponding fish as well as to control fish (0 µg/g). The normalized averages were then plotted against C-dots dosages to determine the magnitude of deposition. Based on three independent experiments, there is a linear relationship between C-dots dosage and photoluminescence intensity (Figure 2(L); linear regression, *R*^2^ > 0.96; Standard t-test, df = 3, *p* < 0.002). Trendlines did not plateau, suggesting that tissue saturation was not achieved. Together, these results suggest that concentrations of C-dots ranging from 5–40 µg/g can label regenerating bone without compromising the fish’s health or the process of bone regeneration. Due to concern that large volumes of C-dots may cause tissue damage during injection, we limited the concentration of C-dots to 20 µg/g in subsequent experiments.

**Figure 3.**
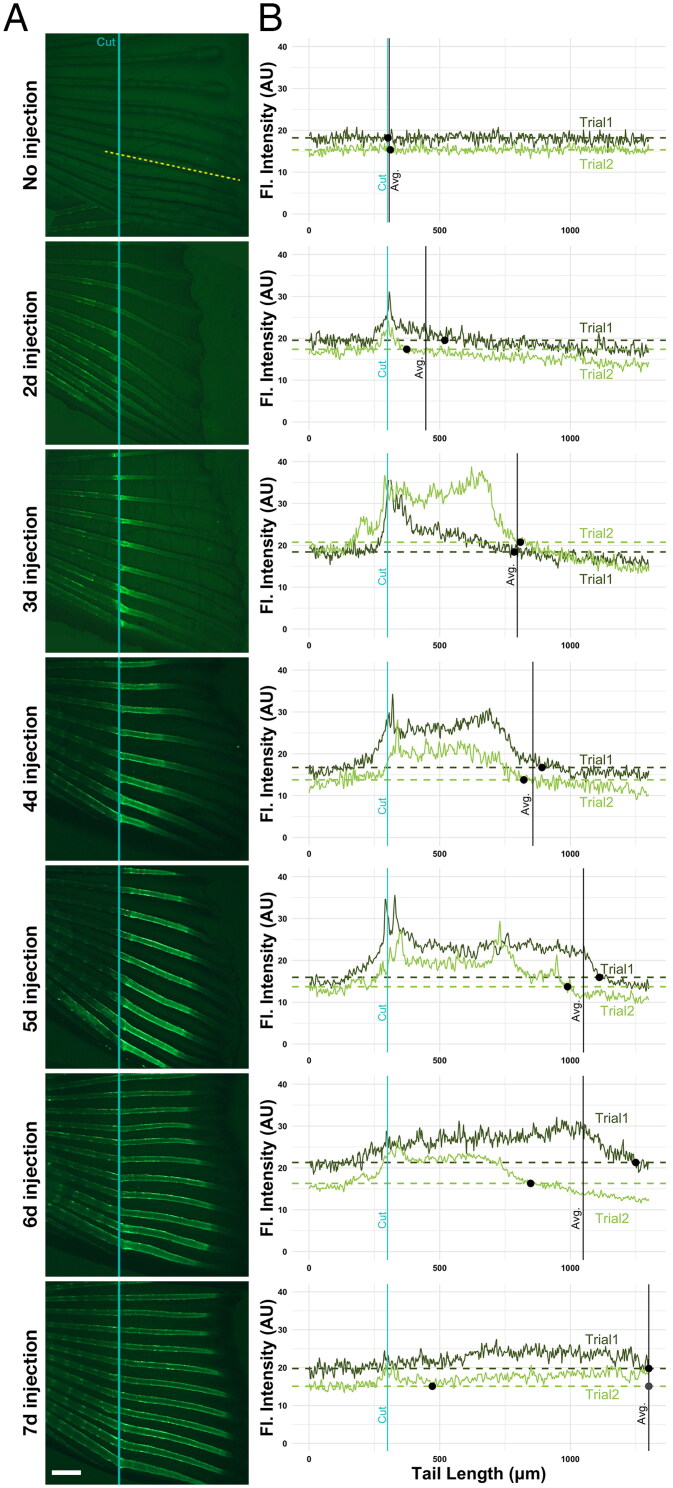
C-dots distribute evenly throughout available regenerating bones. (A) C-dots deposit on available regenerating bone at time of injection. Injection days after amputation are indicated on the left. Specimens were imaged 8 days after amputation, anterior to the left. Site of the cut is indicated with a blue line, and the site of the profile intensity analysis with a yellow dashed line. Experiments were done in duplicate, with three fish per trial. Scale bar is 500 µm. (B) Fluorescent intensity quantification profile across the length of the fourth ventral fin ray (bone), in two representative fish from two independent experiments. Fluorescent signal in arbitrary units (A. U.) was normalized to saline-injected controls. Blue line indicates the site of amputation, black dots indicate the position where fluorescence in regenerating bone reaches average background fluorescence levels (non-regenerated portion of the bone; dashed line), and black line indicates the position where average fluorescence levels in both specimens reach background levels (except in 7-day fish, were average for trial 2 fish reaches background levels twice; gray dot).

Next, to determine whether C-dots deposition on bones is dependent on the state of regeneration, we repeated the experiment varying the time of injection after amputation. The time of C-dot injection varied from 2 to 7 dpa, and all the regenerated fins were imaged at 8 dpa (Figure 3(A)). We then quantified the photoluminescence intensity profile of the C-dots deposited in the fourth most ventral regenerated fin ray (Figure 3(B)). Compared to controls, injection at 2 dpa resulted in the least amount of C-dots deposition, which was primarily restricted to the site of amputation. Injection at subsequent days increased the area of C-dots deposition along regenerating bones, with the whole regenerated ray being labeled at 7 dpa (Figure 3(A)). Signal quantification of the labeled bones revealed an inverse correlation between the amount of tissue labeled and the photoluminescence intensity of the labeling: the more area labeled, the lower the intensity of the photoluminescent signal (Figure 3(B)). These observations suggest that C-dots deposition after fin amputation occurs at all stages of bone regeneration, resulting in a homogeneous distribution of fluorescent labeling throughout the bone tissue distal to the amputation site. Clearance of C-dots from circulation was swift, as C-dots deposition occurred in a regenerated bone already present in the fin, and not in bone regenerated in subsequent days. For example, injection at 3 dpa only labeled the bone that had regenerated at that point, and not the bone that regenerated between 4–7 dpa (Figure 3). Together these results suggest that C-dots have a short circulatory half-life, depositing in available bones at the time of injection.

### C-dots deposit at areas of appositional bone growth

**Figure 4.**
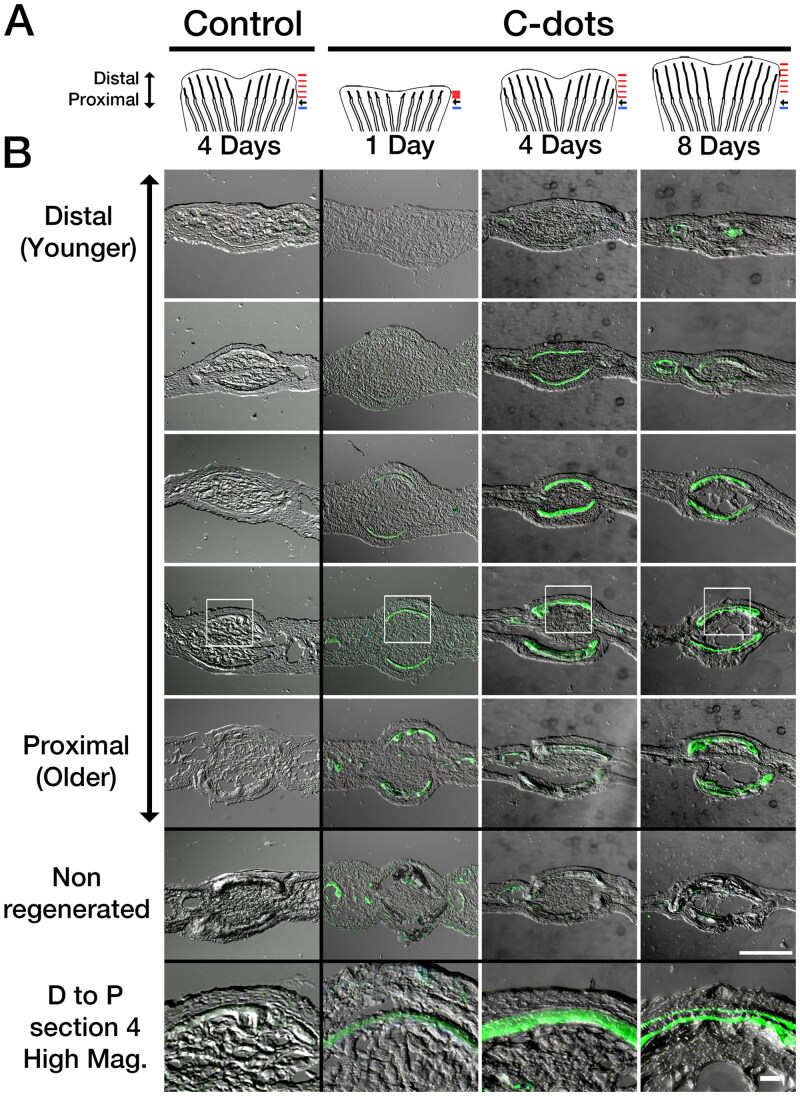
Differential spatiotemporal deposition of C-dots in regenerating bones. (A) Diagram of regenerating fins used in analysis. Amputated fish were injected with saline (control) or C-dots at the indicated times. One hour after injection, fins were re-amputated and processed for cryosectioning. Black arrow indicates amputation site. Red and blue lines indicate the approximate site of sections in regenerating and non-regenerating bones, respectively. (B) Sagittal sections of regenerated and non-regenerated caudal fins ordered in distal (younger) and proximal (older) direction (position corresponding to red and blue lines in A, respectively). Areas of C-dot deposition are green. Areas in white box are shown magnified in the bottom panels. Scale bar is 100 µm for low and 20 µm for high magnification images.

To investigate the dynamics of C-dot deposition at the tissue level, we analyzed the distribution of C-dots’ photoluminescence in transverse sections of regenerating caudal fins. Adult zebrafish were injected with C-dots 1, 4 or 8 dpa and, after 1-h of labeling, the fin was harvested, cryosectioned, and imaged using confocal microscopy. We did not observe any histological differences in the regenerated bones between control and C-dot injected fish (Figure 4 and data not shown), supporting the gross morphological observations that C-dots deposition does not interfere with bone regeneration processes. At all stages of regeneration, bones proximal to the amputation site were thicker, more mineralized, and exhibited a strong C-dot signal (Figure 4(B)). To test if bone age is a determinant of C-dot deposition, we analyzed cross-sections of bones at different stages of regeneration at comparable proximal-distal positions. Comparison of representative sections adjacent to the amputation site revealed weak labeling of newly regenerated bones at 1 dpa, strong and even labeling at 4 dpa, and strong labeling of bone surfaces at 8 dpa (Figure 4). Given that the diameter of bones increases by the repeated addition of ossified tissue at the surface (appositional growth (Rauch, 2005)), our observations suggest that C-dot deposition occurs in areas of bone growth.

**Figure 5.**
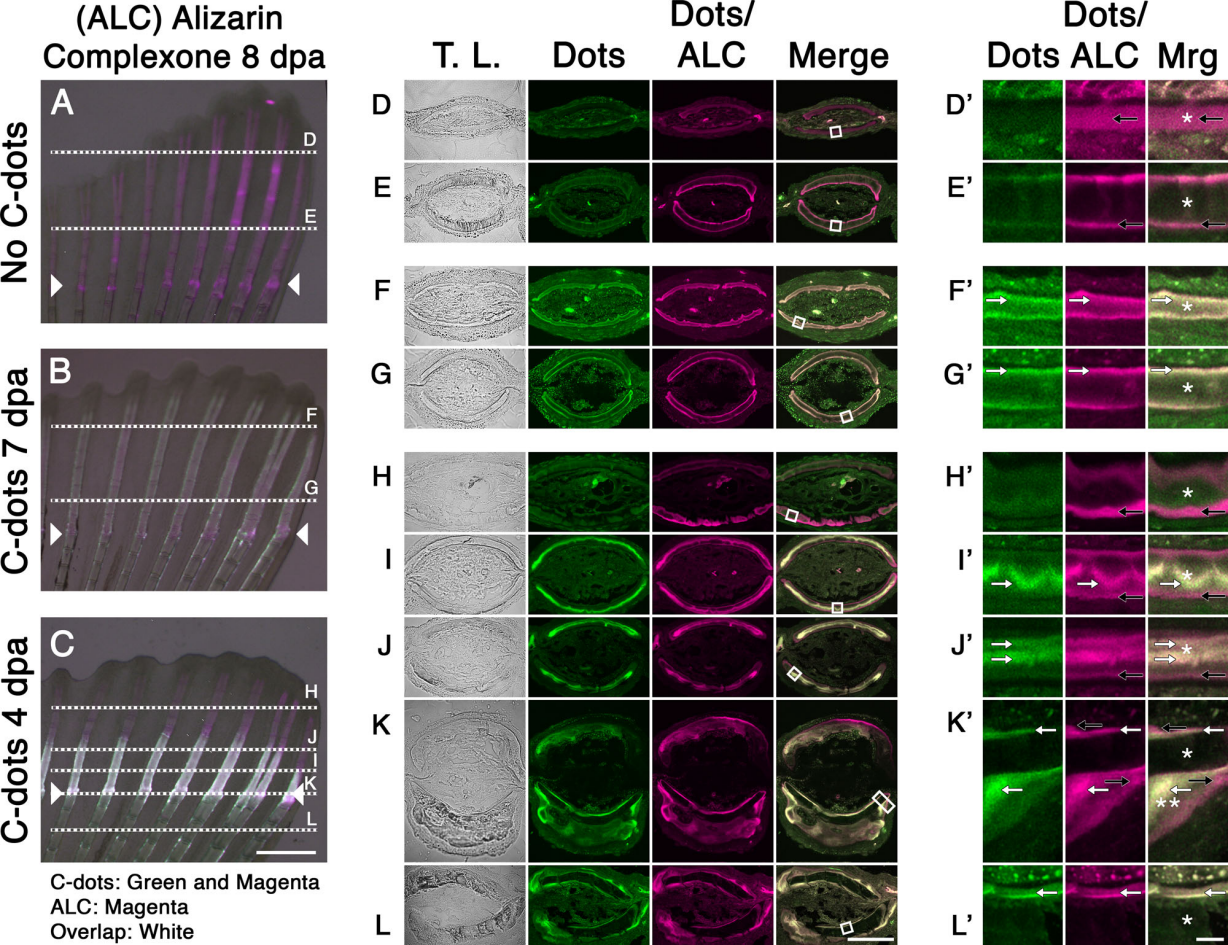
C-dots deposit at sites of active bone mineralization. (A–C) Regenerated caudal fin of uninjected fish (A), injected with C-dots at 7 dpa (B), or injected at 4 dpa (C). All fish were stained with Alizarin Complexone (ALC) at 8 dpa and imaged. Composite images were generated by superimposing photographs obtained using transmitted light, a 488/525 nm filter set to capture C-dots’ fluorescence (pseudo colored green), and a 596/615 nm filter set to capture C-dots/ALC’s fluorescence (pseudo colored magenta). ALC-positive areas (indicative of ossification) that lack C-dots appear magenta, and areas of ossification with C-dot deposition appear white. Posterior end of fin toward top of images. Site of amputation is indicated with arrowheads and the plane of sections (D)–(L) are indicated with dotted lines. (D–L) Transverse sections of fins, imaged and pseudo colored as in (A)–(C). (D’–L’) Magnified bone regions from D–E (white squares). Areas of C-dot deposition are indicated with white arrows. Ossified regions devoid of C-dots are indicated with black arrows. Asterisks indicate the bone matrix. Double asterisk in K’ indicates areas of matrix buildup at the site of amputation. Scale bars are 500 µm for (A)–(C), 50 µm for (D)–(L) and 5 µm for (D’)–(L’).

To directly test C-dots deposition in areas of tissue growth, we examined the distribution of C-dots relative to that of the dye Alizarin Red Complexone (ARC). Intravital staining of bones by Alizarin red dyes is a well-established histological method used in mice to distinguish areas of active bone growth, as the dye is preferentially taken up by these areas compared to regions where bone growth has slowed down or ceased (Hoyte, 1960). Thus, co-localization of C-dots and ALC signals would indicate that C-dots preferentially bind to areas of active bone growth. To validate the use of ALC in zebrafish, we exposed fish undergoing caudal fin regeneration to ALC at 8 dpa. In these fish, regenerated fin bones were strongly stained with ALC (Figure 5(A)). In cross-sections, ALC’s stain distribution was homogeneous in bones closer to the fin tips (Figure 5(D,D’)), and superficial in bones closer to the amputation site (Figure 5(E,E’)). These findings are similar to those observed during the normal growth of bones in mammals (Hoyte, 1960), supporting the use of ALC as a viable method for identifying areas of bone growth in zebrafish.

Next, we investigated the relationship between C-dot deposition and areas of ALC staining. This analysis, however, had to be done indirectly, as C-dots’ 500–600 nm emission range overlaps with the emission peak of ALC at 580 nm. Using an exclusion approach, we could identify areas of growth that were ALC-positive and C-dots-negative by looking for 580 nm fluorescence in the absence of 525 fluorescence. We began this analysis by injecting fish with C-dots at 7 dpa and staining them with ALC one day later (Figure 5(B)). Because bone layer formation takes more than 24 h, we expected to see extensive C-dots and ALC signal co-localization. In all sections examined, the 525 and 580 nm signals co-localized (Figure 5(F,F’,G,G’)). At the distal tips of the fins, signal co-localization in newly formed bones were observed at the core and surface of the tissue (Figure 5(F,F’)), whereas closer to the amputation site, signal co-localization in older bones was observed only at the tissue’s surface (Figure 5(G,G’)). This signal co-localization suggested that C-dots deposition occurred in areas of appositional growth. To further test this idea, we spatially separated temporal areas of growth by injecting fish with C-dots at 4 dpa and staining them with ALC four days later. In this experiment, we hypothesized that areas of early bone growth would fluoresce at 525 and 580 nm indicating C-dots deposits, whereas areas of late bone growth would fluoresce only at 580 nm indicating ALC staining. Consistent with this hypothesis, whole-mount analysis of the fins revealed that regenerated bones had an even distribution of the 580 nm fluorescent signal and a proximally-confined distribution of the 525 nm fluorescent signal (Figure 5(C)). Sectioning of the tissue further confirmed our hypothesis. Bones in distal regions of the fin showed 580 nm fluorescence only at their surface, indicating that areas of new growth stained only with ALC (Figure 5(H,H’)). In contrast, bones at the amputation site or that did not undergo regeneration were positive for both fluorescent signals at the bone surface, indicating C-dots deposits and, presumably, ALC staining (Figure 5(K,K’,L,L’)). Strikingly, between these two regions, bones that were in the process of regeneration at the time of C-dot injection showed a dual staining pattern: the core portion of the bone was positive for both fluorescent signals indicative of C-dot deposits, while the surface of the bone was only positive for the 580 nm signal indicative of ALC staining (Figure 5(I,I’,J,J’)). We interpret this dual staining pattern to indicate that, as regenerating bones increase in size due to the apposition of new layers of tissue at their surface, early deposits of C-dots become surrounded by new tissue that stains positive for ALC. This observation, together with our previous result showing that C-dots and ALC signals overlap when treatments are done within 24 h of each other suggests that C-dots bind to areas of bone growth.

## Conclusions

The discovery that C-dots bind to areas of adult bone growth without interfering with the biological processes of homeostatic turnover, repair, and regeneration opens the possibility for their use as theragnostic agents for diseases such as osteoporosis. Current osteoporosis treatments rely on early diagnosis to mitigate further bone erosion, as limited treatments exist to restore lost bone mass. C-dots’ intrinsic photoluminescence and bone-binding properties could revolutionize osteoporosis diagnostic tools by allowing clear visualization of the active and inactive areas of bone growth. For treatment, C-dots could be used as fluorescently traceable, bone-specific, drug-delivery agents. We have previously shown that surface modification of C-dots with ethylenediamine or glutamic acid does not result in loss of fluorescence or bone binding properties in larvae, providing proof of principle of their possible use as a bone-specific drug delivery method (Peng et al., 2017). Future work is needed to establish that drug conjugation to C-dots does not result in loss of the drug’s biological activity. Together, our results provide an important foundation for future applications of C-dots as a drug delivery vehicle for the treatment of adult skeletal diseases and traumatic bone injuries.

## Supplementary Material

Supplemental Material
