# Supplementary material for: In vivo characterization of carbon dots–bone interactions: toward the development of bone-specific nanocarriers for drug delivery
Source: Drug Deliv. 2021 Jun 26;28(1):1281–9. doi: 10.1080/10717544.2021.1938753 (PMC8238062; doi:10.1080/10717544.2021.1938753)
Supplement: Supplemental Material [file IDRD_A_1938753_SM6812.pdf]

Figure 3 - C-dots Distributed Along Regernating Bone

```
regeneration <- read_csv("/Users/racheldumez/OneDrive - University of North Carolin
a at Chapel Hill/Attachments/STAT209 Lab files/regenerationtimelineround1.csv")
```

```
## Warning: Missing column names filled in: 'X16' [16]
```

```
## Parsed with column specification:
## cols(
##   length = col_double(),
##   two_dpc = col_double(),
##   three_dpc = col_double(),
##   four_dpc = col_double(),
##   five_dpc = col_double(),
##   six_dpc = col_double(),
##   seven_dpc = col_double(),
##   control = col_double(),
##   two_dpc_2 = col_double(),
##   three_dpc_2 = col_double(),
##   four_dpc_2 = col_double(),
##   five_dpc_2 = col_double(),
##   six_dpc_2 = col_double(),
##   seven_dpc_2 = col_double(),
##   control_2 = col_double(),
##   X16 = col_logical(),
##   three_dpc_rollingwindow = col_double(),
##   three_dpc2_rollingwindow = col_double()
## )
```

```
ggplot(regeneration, aes(length, two_dpc)) +
  geom_line(color = "darkolivegreen") +
  geom_hline(yintercept=19.52100259, linetype="dashed", color = "darkolivegreen", siz
e = .8) +
  geom_line(aes(y=two_dpc_2), color = "darkolivegreen3") +
  geom_hline(yintercept = 17.369725, color="darkolivegreen3", linetype="dashed",size=
.8) +
  #xlab("Tail Length (μm)") + # how to make this a dif color theme(axis...)
  ylab("Fl. Intensity (AU)") +
  theme(axis.title = element_text(size = 18, face = "bold", color = "black")) +
  #ggtitle("Injection At Two Days Post Amputation") +  # how to put it in the middle
  geom_vline(xintercept = 300, color="cyan3",size=.5) +
  geom_vline(xintercept = 446.739, color="black",size=.5) +
  annotate("text", x = 280, y = 12.6, label = "Cut", color = "cyan3", size = 4, angle
= 90) +
  annotate("text", x = 420, y = 13, label = "Avg.", color = "black", size = 4, angle=
90) +
  annotate("text", x = 1100, y = 11.5, label = "Trial2", color = "darkolivegreen3", s
ize = 5, face = "bold") +
  annotate("text", x = 1100, y = 22.6, label = "Trial1", color = "darkolivegreen", si
ze = 5, face = "bold") +
  annotate("point", x =519.565 , y = 19.52100259, color = "black", size = 3) +
  annotate("point", x =373.913 , y = 17.369725, color = "black", size = 3) +
  theme(plot.title = element_text(hjust = 0.5),axis.title.x = element_text(colour = "
black"),axis.title.y = element_text(colour = "black")) +
  ylim(0, 40) +
  theme(axis.title.x = element_blank()) +
  theme(axis.text.x = element_text(face="bold", color = "black"),axis.text.y = elemen
t_text(face="bold",color = "black"))
```

```
## Warning: Ignoring unknown parameters: face

## Warning: Ignoring unknown parameters: face
```

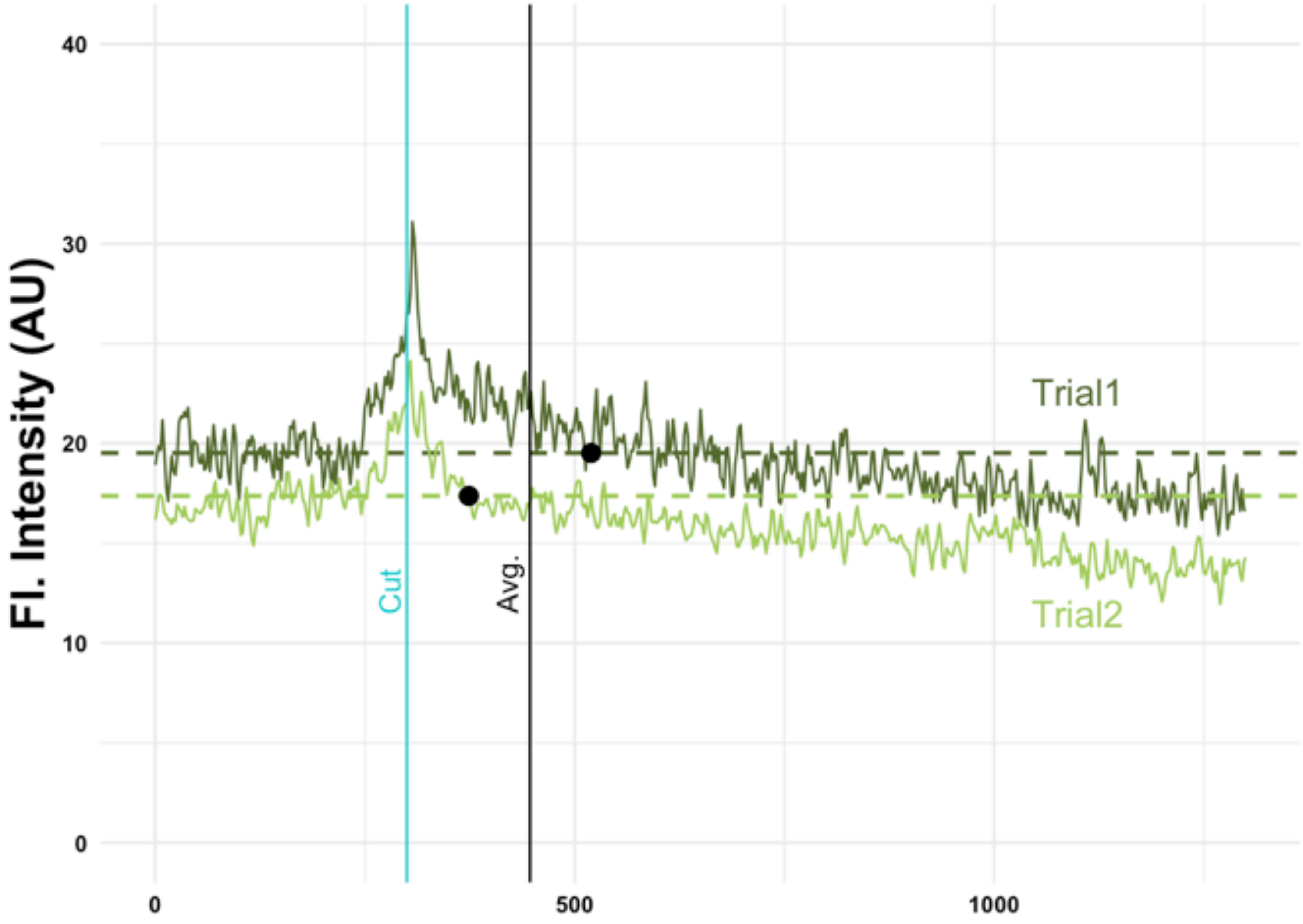


```
#ggsave("Injection at two days post amputation final_withavgline_ylim40.jpeg", height
= 3, width = 6)
```

```
#filter(regeneration, two_dpc <21.81745, length >300) $length[1]
```

```
#filter(regeneration, two_dpc_2 <17.369725, length >300) $length[1]
```

```
regenerationrollingaverage <- read_csv("/Users/racheldumez/OneDrive - University of N
orth Carolina at Chapel Hill/Attachments/STAT209 Lab files/regenerationtimelineround1
rollingwindow.csv")
```

```
## Parsed with column specification:
## cols(
##   length = col_double(),
##   two_dpc_rollingwindow = col_double(),
##   two_dpc2_rollingwindow = col_double(),
##   three_dpc_rollingwindow = col_double(),
##   three_dpc2_rollingwindow = col_double(),
##   four_dpc_rollingwindow = col_double(),
##   four_dpc2_rollingwindow = col_double(),
##   five_dpc_rollingwindow = col_double(),
##   five_dpc2_rollingwindow = col_double(),
##   six_dpc_rollingwindow = col_double(),
##   six_dpc2_rollingwindow = col_double(),
##   seven_dpc_rollingwindow = col_double(),
##   seven_dpc2_rollingwindow = col_double(),
##   control_rollingwindow = col_double(),
##   Control_2_rollingwindow = col_double()
## )
```

```
filter(regenerationrollingaverage, two_dpc_rollingwindow <19.52100259, length >300) $
length[1]
```

```
## [1] 519.565
```

```
filter(regenerationrollingaverage, two_dpc2_rollingwindow <17.369725, length >300) $l
ength[1]
```

```
## [1] 373.913
```

```
ggplot(regeneration, aes(length, three_dpc)) +
  geom_line(color = "darkolivegreen") +
  geom_hline(yintercept=18.41830765, linetype="dashed", color = "darkolivegreen", siz
e = .8) +
  geom_line(aes(y=three_dpc_2), color = "darkolivegreen3") +
  geom_hline(yintercept = 20.74863956, color="darkolivegreen3", linetype="dashed",siz
e=.8) +
  ylab("Fl. Intensity (AU)") +
  theme(axis.title = element_text(size = 18, face = "bold", color = "black")) +
  geom_vline(xintercept = 300, color="cyan3",size=.5) +
  geom_vline(xintercept = 796.7395, color="black",size=.5) +
  annotate("text", x = 280, y = 12.5, label = "Cut", color = "cyan3", size = 4, angle
= 90) +
  annotate("text", x = 770, y = 13, label = "Avg.", color = "black", size = 4, angle=
90) +
  annotate("text", x = 1100, y = 23, label = "Trial2", color = "darkolivegreen3", siz
e = 5, face = "bold") +
  annotate("text", x = 1100, y = 12, label = "Trial1", color = "darkolivegreen", size
= 5, face = "bold") +
  annotate("point", x =784.783 , y = 18.41830765, color = "black", size = 3) +
  annotate("point", x =808.696 , y = 20.74863956, color = "black", size = 3) +
  theme(plot.title = element_text(hjust = 0.5),axis.title.x = element_text(colour = "
black"),axis.title.y = element_text(colour = "black")) +
  theme(axis.title.x = element_blank()) +
  ylim(0, 40) +
  theme(axis.title.x = element_blank()) +
  theme(axis.text.x = element_text(face="bold", color = "black"),axis.text.y = elemen
t_text(face="bold",color = "black"))
```

```
## Warning: Ignoring unknown parameters: face

## Warning: Ignoring unknown parameters: face
```

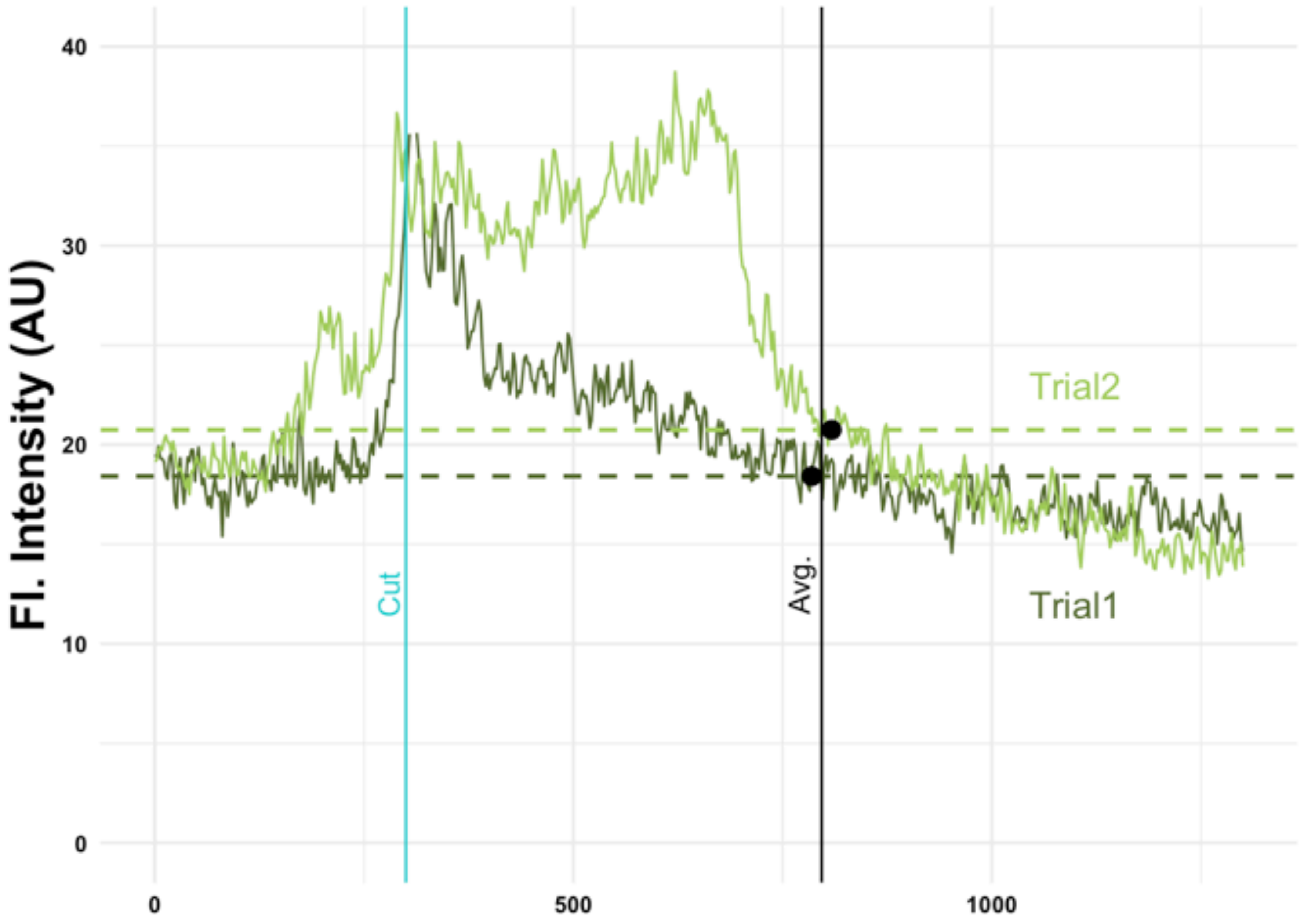


```
#ggsave("Injection At Three Days Post Amputation_ylim40_final_withavgline.jpeg", heig
ht = 3, width = 6)
```

```
#filter(regeneration, three_dpc <19.08573, length >300) $length[1]
```

```
#filter(regeneration, three_dpc_2 <23.64883, length >300) $length[1]
```

```
filter(regenerationrollingaverage, three_dpc_rollingwindow <18.41830765, length >300)
$length[1]
```

```
## [1] 784.783
```

```
filter(regenerationrollingaverage, three_dpc2_rollingwindow <20.74863956, length >300
) $length[1]
```

```
## [1] 808.696
```

```
ggplot(regeneration, aes(length, four_dpc)) +
  geom_line(color = "darkolivegreen") +
  geom_hline(yintercept=16.71893922, linetype="dashed", color = "darkolivegreen", siz
e = .8) +
  geom_line(aes(y=four_dpc_2), color = "darkolivegreen3") +
  geom_hline(yintercept = 13.77505872, color="darkolivegreen3", linetype="dashed",siz
e=.8) +
  ylab("Fl. Intensity (AU)") +
  theme(axis.title = element_text(size = 18, face = "bold", color = "black")) +
  geom_vline(xintercept = 300, color="cyan3",size=.5) +
  geom_vline(xintercept = 856.5215, color="black",size=.5) +
  annotate("text", x = 280, y = 7.5, label = "Cut", color = "cyan3", size = 4, angle=
90) +
  annotate("text", x = 827, y = 8, label = "Avg.", color = "black", size = 4, angle=
90) +
  annotate("text", x = 1100, y = 8.5, label = "Trial2", color = "darkolivegreen3", si
ze = 5, face = "bold") +
  annotate("text", x = 1100, y = 18.7, label = "Trial1", color = "darkolivegreen", si
ze = 5, face = "bold") +
  annotate("point", x =891.304 , y = 16.718939223, color = "black", size = 3) +
  annotate("point", x = 821.739 , y = 13.77505872, color = "black", size = 3) +
  theme(plot.title = element_text(hjust = 0.5),axis.title.x = element_text(colour = "
gray26"),axis.title.y = element_text(colour = "black")) +
  theme(axis.title.x = element_blank()) +
  ylim(0, 40) +
  theme(axis.title.x = element_blank()) +
  theme(axis.text.x = element_text(face="bold", color = "black"),axis.text.y = elemen
t_text(face="bold",color = "black"))
```

```
## Warning: Ignoring unknown parameters: face

## Warning: Ignoring unknown parameters: face
```

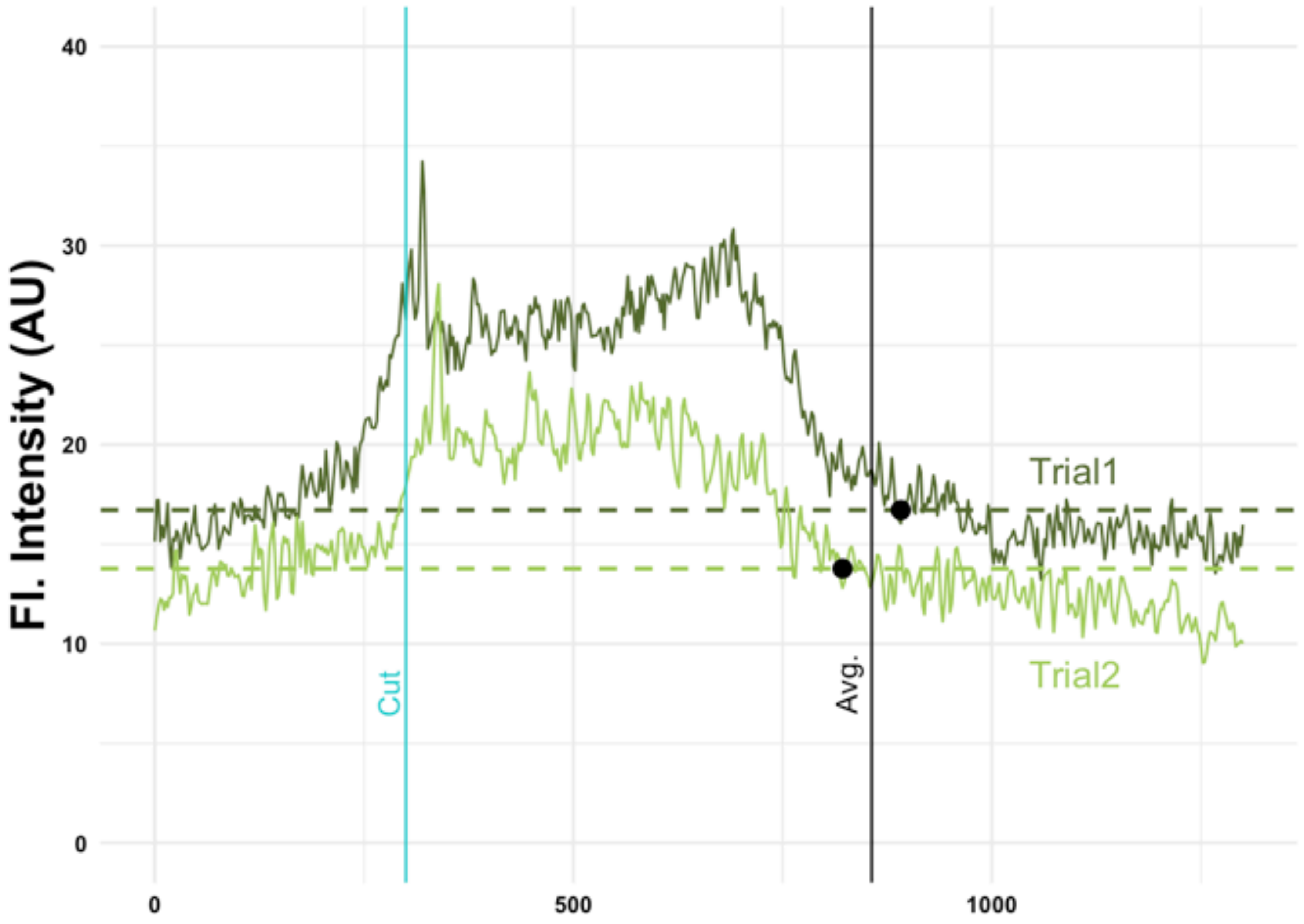


```
#ggsave("Injection At Four Days Post Amputation_ylim40_withavgline_final.jpeg", heigh
t = 3, width = 6)
```

```
#filter(regeneration, four_dpc <20.45543333, length >300) $length[1]
```

```
#filter(regeneration, four_dpc_2 <14.8414, length >300) $length[1]
```

```
filter(regenerationrollingaverage, four_dpc_rollingwindow <16.71893922, length >300)
$length[1]
```

```
## [1] 891.304
```

```
filter(regenerationrollingaverage, four_dpc2_rollingwindow <13.77505872, length >300)
$length[1]
```

```
## [1] 821.739
```

```
ggplot(regeneration, aes(length, five_dpc)) +
  geom_line(color = "darkolivegreen") +
  geom_hline(yintercept=15.950061873, linetype="dashed", color = "darkolivegreen", size = .8) +
  geom_line(aes(y=five_dpc_2), color = "darkolivegreen3") +
  geom_hline(yintercept = 13.71111643, color="darkolivegreen3", linetype="dashed",size=.8) +
  ylab("Fl. Intensity (AU)") +
  theme(axis.title = element_text(size = 18, face = "bold", color = "black")) +
  geom_vline(xintercept = 300, color="cyan3",size=.5) +
  geom_vline(xintercept = 1049.965, color="black",size=.5) +
  annotate("text", x = 280, y = 7.5, label = "Cut", color = "cyan3", size = 4, angle= 90) +
  annotate("text", x = 1020.5, y = 8, label = "Avg.", color = "black", size = 4, angle= 90) +
  annotate("text", x = 1180, y = 8, label = "Trial2", color = "darkolivegreen3", size = 5) +
  annotate("text", x = 1180, y = 18, label = "Trial1", color = "darkolivegreen", size = 5) +
  annotate("point", x =1110.87 , y = 15.950061873, color = "black", size = 3) +
  annotate("point", x =989.13 , y = 13.71111643, color = "black", size = 3) +
  theme(plot.title = element_text(hjust = 0.5),axis.title.x = element_text(colour = "black"),axis.title.y = element_text(colour = "black")) +
  ylim(0, 40) +
  theme(axis.title.x = element_blank()) +
  theme(axis.text.x = element_text(face="bold", color = "black"),axis.text.y = element_text(face="bold",color = "black"))
```

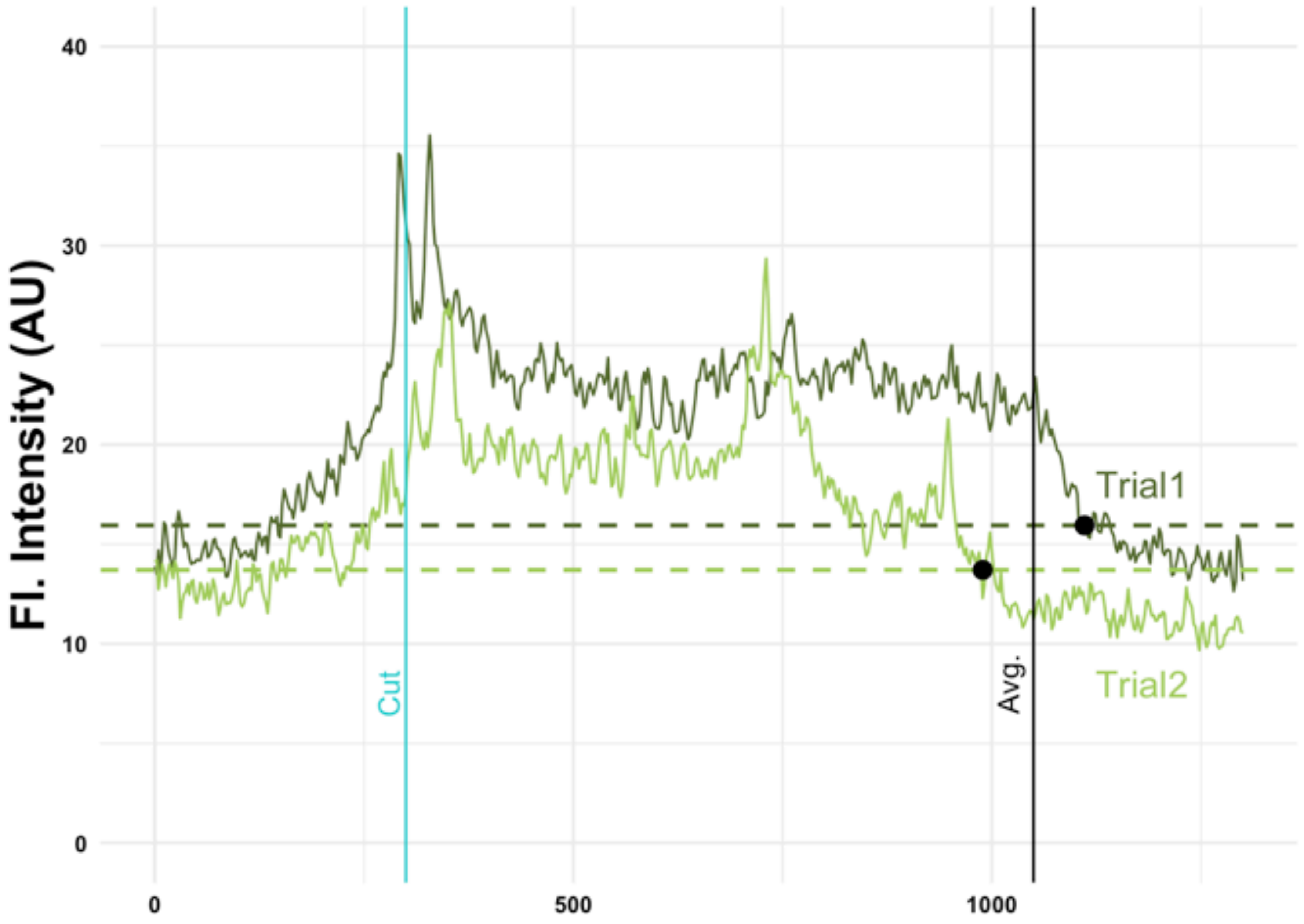


```
#ggsave("Injection At Five Days Post Amputation_ylim40_withavgline_final.jpeg", heigh
t = 3, width = 6)
```

```
#filter(regeneration, five_dpc <20.2, length >300) $length[1]
```

```
#filter(regeneration, five_dpc_2 <15.727, length >300) $length[1]
```

```
filter(regenerationrollingaverage, five_dpc_rollingwindow <15.950061873, length >300)
$length[1]
```

```
## [1] 1110.87
```

```
filter(regenerationrollingaverage, five_dpc2_rollingwindow <13.71111643, length >300)
$length[1]
```

```
## [1] 989.13
```

```
ggplot(regeneration, aes(length, six_dpc)) +
  geom_line(color = "darkolivegreen") +
  geom_hline(yintercept=21.29430729, linetype="dashed", color = "darkolivegreen", siz
e = .8) +
  geom_line(aes(y=six_dpc_2), color = "darkolivegreen3") +
  geom_hline(yintercept = 16.29928549, color="darkolivegreen3", linetype="dashed",siz
e=.8) +
  ylab("Fl. Intensity (AU)") +
  theme(axis.title = element_text(size = 18, face = "bold", color = "black")) +
  geom_vline(xintercept = 300, color="cyan3",size=.5) +
  geom_vline(xintercept = 1048.913, color="black",size=.5) +
  annotate("text", x = 280, y = 7.5, label = "Cut", color = "cyan3", size = 4, angle=
90) +
  annotate("text", x = 1020, y = 8, label = "Avg.", color = "black", size = 4, angle=
90) +
  annotate("text", x = 1160, y = 8, label = "Trial2", color = "darkolivegreen3", size
= 5, face = "bold") +
  annotate("text", x = 1160, y = 32, label = "Trial1", color = "darkolivegreen", size
= 5, face = "bold") +
  annotate("point", x = 1250 , y = 21.29430729, color = "black", size = 3) +
  annotate("point", x = 847.826 , y = 16.29928549, color = "black", size = 3) +
  theme(plot.title = element_text(hjust = 0.5),axis.title.x = element_text(colour = "
black"),axis.title.y = element_text(colour = "black")) +
  ylim(0, 40) +
  theme(axis.title.x = element_blank()) +
  theme(axis.text.x = element_text(face="bold", color = "black"),axis.text.y = elemen
t_text(face="bold",color = "black"))
```

```
## Warning: Ignoring unknown parameters: face

## Warning: Ignoring unknown parameters: face
```

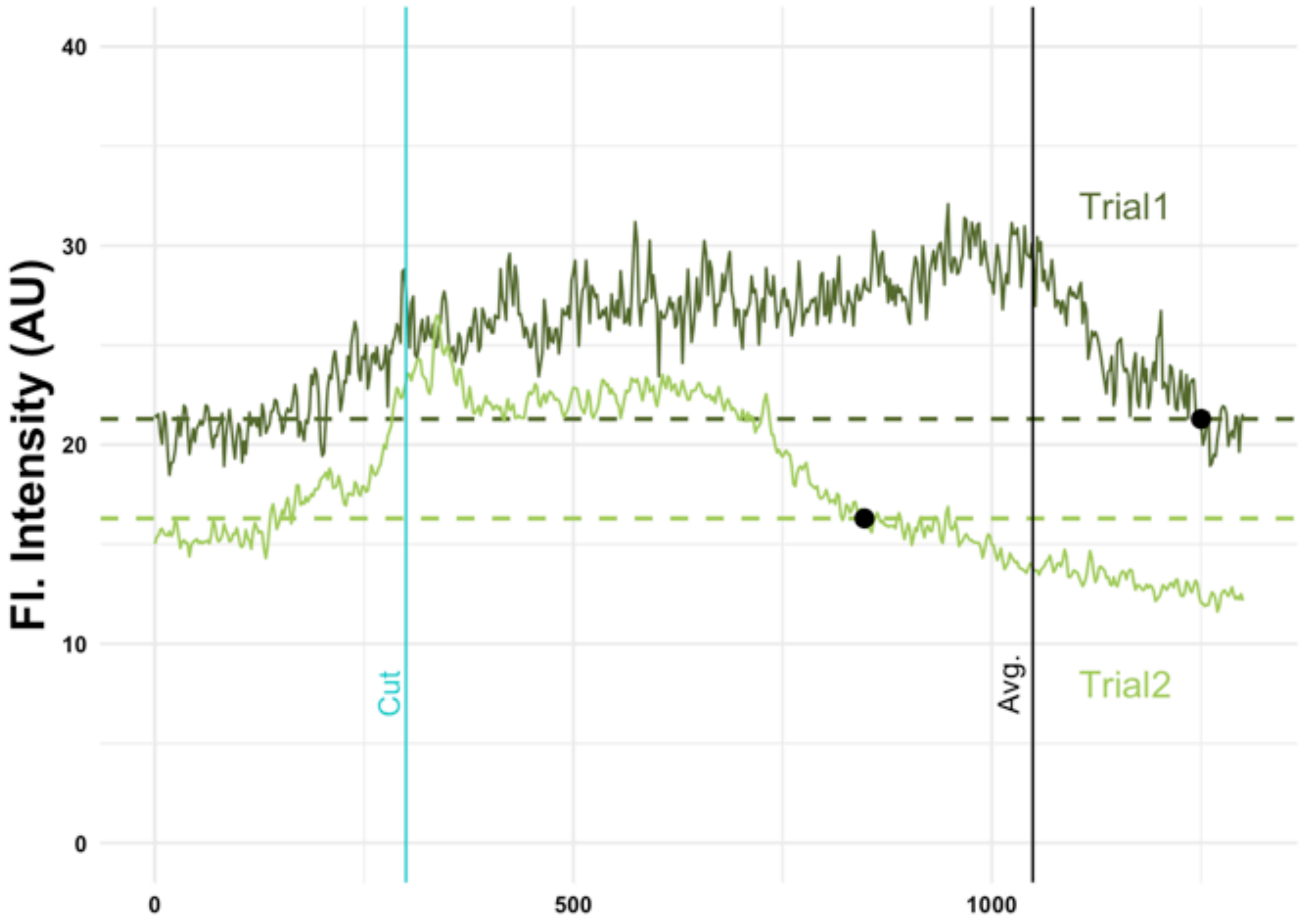


```
#ggsave("Injection At Six Days Post Amputation_ylim40_withavgline_final.jpeg", height
= 3, width = 6)
```

```
#filter(regeneration, six_dpc <21.29430729, length >300) $length[1]
```

```
#filter(regeneration, six_dpc_2 <16.29928549, length >300) $length[1]
```

```
filter(regenerationrollingaverage, six_dpc_rollingwindow <21.29430729, length >300) $
length[1]
```

```
## [1] 1250
```

```
filter(regenerationrollingaverage, six_dpc2_rollingwindow <16.29928549, length >300)
$length[1]
```

```
## [1] 847.826
```

```
ggplot(regeneration, aes(length, seven_dpc)) +
  geom_line(color = "darkolivegreen") +
  geom_hline(yintercept=19.76384556, linetype="dashed", color = "darkolivegreen", size = .8) +
  geom_line(aes(y=seven_dpc_2), color = "darkolivegreen3") +
  geom_hline(yintercept = 15.08218625, color="darkolivegreen3", linetype="dashed",size=.8) +
  xlab("Tail Length (µm)") +
  ylab("Fl. Intensity (AU)") +
  theme(axis.title = element_text(size = 18, face = "bold", color = "black")) +
  geom_vline(xintercept = 300, color="cyan3",size=.5) +
  geom_vline(xintercept = 1300, color="black",size=.5) +
  annotate("text", x = 280, y = 7.5, label = "Cut", color = "cyan3", size = 4, angle=90) +
  annotate("text", x = 1272, y = 7.9, label = "Avg.", color = "black", size = 4, angle= 90) +
  annotate("text", x = 1122, y = 13, label = "Trial2", color = "darkolivegreen3", size = 5, face = "bold") +
  annotate("text", x = 1122, y = 27.5, label = "Trial1", color = "darkolivegreen", size = 5, face = "bold") +
  annotate("point", x =1300 , y = 19.76384556, color = "black", size = 3) +
  annotate("point", x = 471.739 , y = 15.08218625, color = "black", size = 3) +
  annotate("point", x = 1300 , y = 15.08218625, color = "gray35",size = 3) +
  theme(plot.title = element_text(hjust = 0.5),axis.title.x = element_text(colour = "black"),axis.title.y = element_text(colour = "black")) +
  ylim(0, 40) +
  theme(axis.text.x = element_text(face="bold", color = "black"),axis.text.y = element_text(face="bold",color = "black"))
```

```
## Warning: Ignoring unknown parameters: face

## Warning: Ignoring unknown parameters: face
```

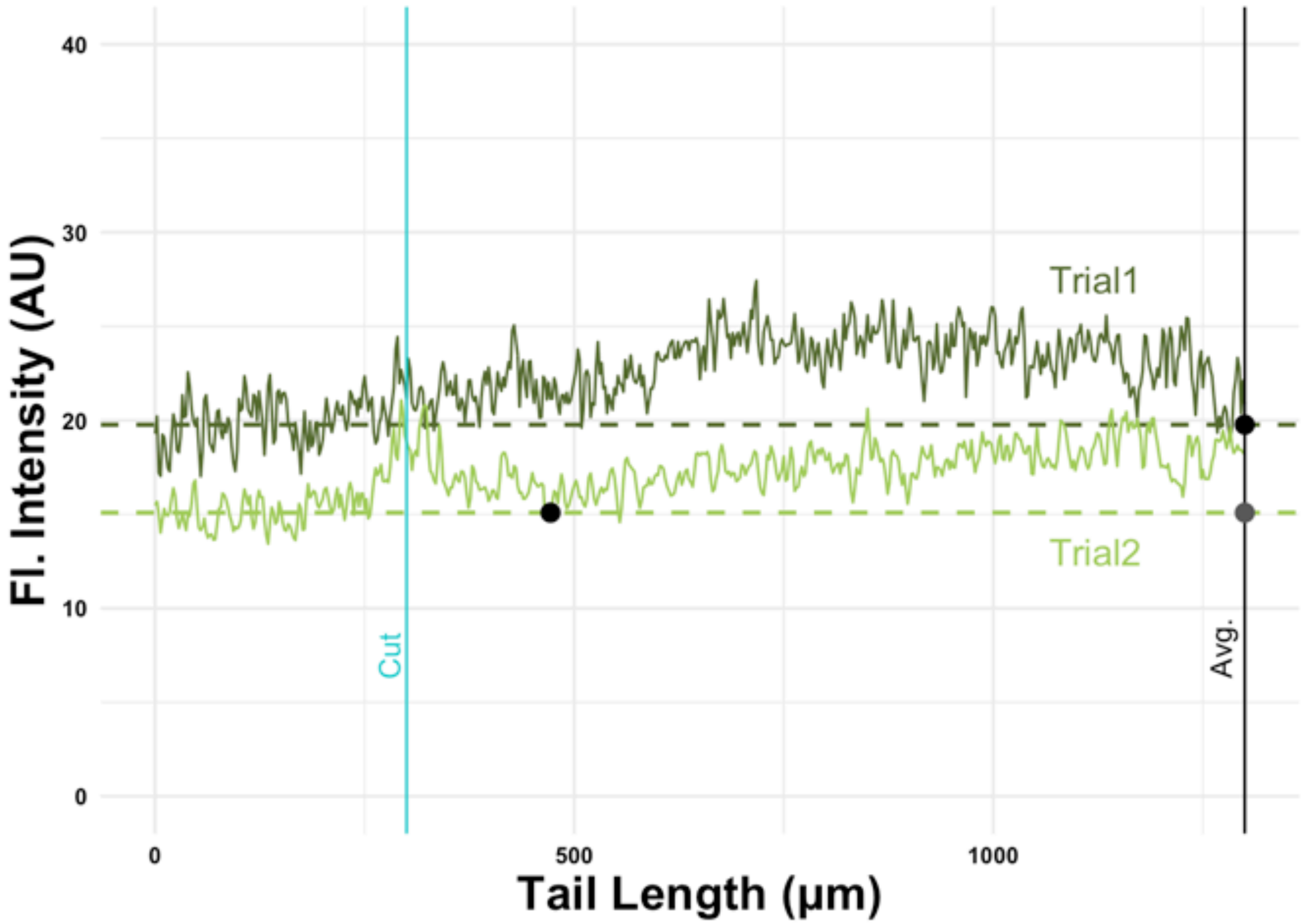


```
#ggsave("Injection At Seven Days Post Amputation_ylim40_withavgline_final.jpeg", heig
ht = 3, width = 6)
```

```
filter(regeneration, seven_dpc <19.76384556, length >600) $length[1]
```

```
## [1] 1267.391
```

```
filter(regeneration, seven_dpc_2 <15.08218625, length >300) $length[1]
```

```
## [1] 465.217
```

```
filter(regenerationrollingaverage, six_dpc2_rollingwindow <19.76384556, length >300)
$length[1]
```

```
## [1] 747.826
```

```
filter(regenerationrollingaverage, seven_dpc_rollingwindow <19.76384556, length >300)
$length[1]
```

```
## [1] NA
```

```
filter(regenerationrollingaverage, seven_dpc2_rollingwindow <15.08218625, length >300
) $length[1]
```

```
## [1] 471.739
```

```
ggplot(regeneration, aes(length, control)) +
  geom_line(color = "darkolivegreen") +
  geom_hline(yintercept=18.18944184, linetype="dashed", color = "darkolivegreen", siz
e = .8) +
  geom_line(aes(y=control_2), color = "darkolivegreen3") +
  geom_hline(yintercept = 15.33677786, color="darkolivegreen3", linetype="dashed",siz
e=.8) +
  ylab("Fl. Intensity (AU)") +
  theme(axis.title = element_text(size = 18, face = "bold", color = "black")) +
  geom_vline(xintercept = 300, color="cyan3",size=.4) +
  geom_vline(xintercept = 306.522, color="black",size=.5) +
  annotate("text", x = 280, y = 7.5, label = "Cut", color = "cyan3", size = 4, angle=
90) +
  annotate("text", x = 327, y = 8.1, label = "Avg.", color = "black", size = 4, angle
= 90) +
  annotate("text", x = 1100, y = 12, label = "Trial2", color = "darkolivegreen3", siz
e = 5) +
  annotate("text", x = 1100, y = 22, label = "Trial1", color = "darkolivegreen", size
= 5) +
  annotate("point", x =302.174 , y = 18.18944184, color = "black", size = 3) +
  annotate("point", x = 310.87 , y = 15.33677786, color = "black", size = 3) +
  theme(plot.title = element_text(hjust = 0.5),axis.title.x = element_text(colour = "
black"),axis.title.y = element_text(colour = "black")) +
 ylim(0, 40) +
  theme(axis.title.x = element_blank()) +
  theme(axis.text.x = element_text(face="bold", color = "black"),axis.text.y = elemen
t_text(face="bold",color = "black"))
```

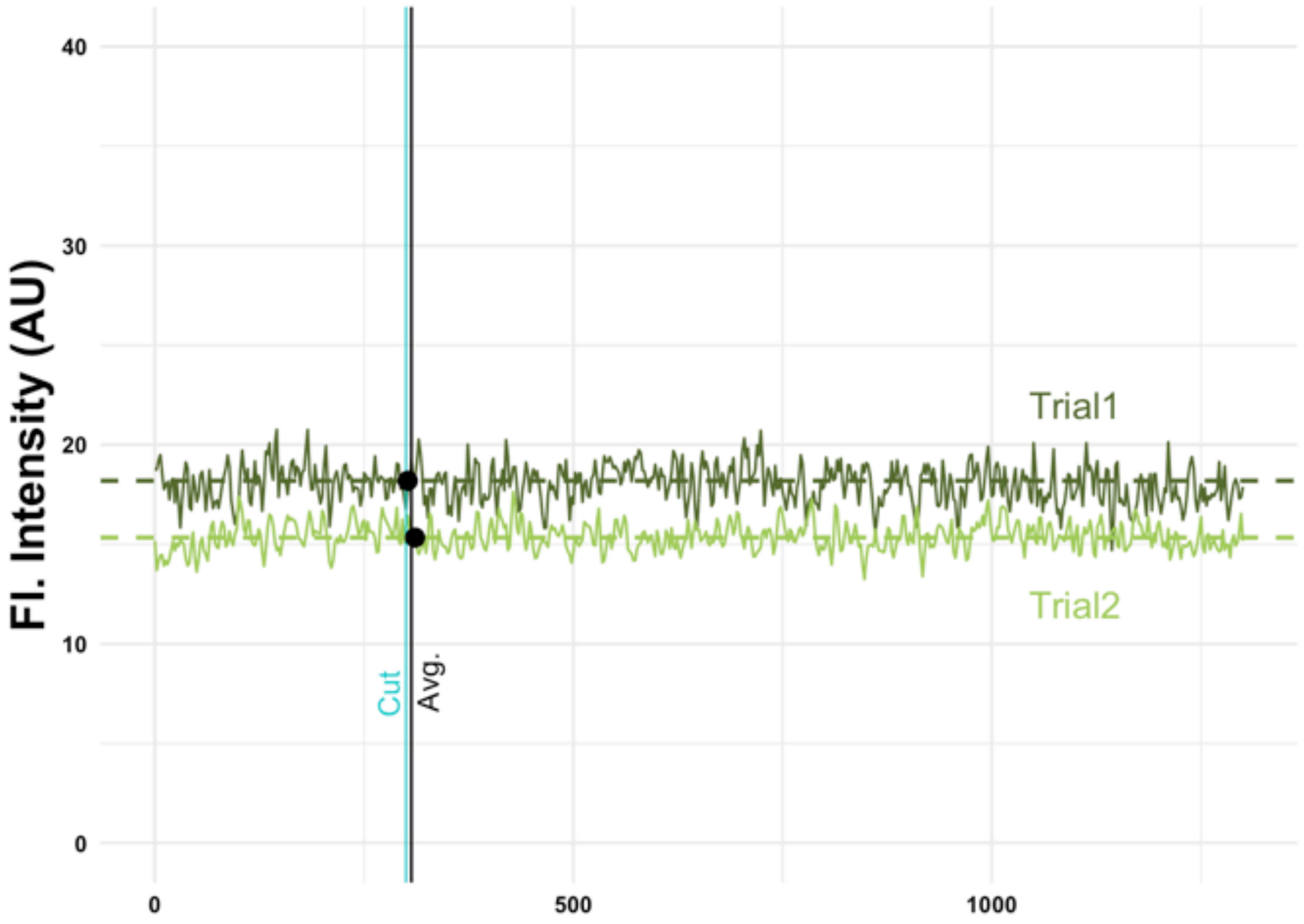


```
ggsave("Control_ylim40_withavgline_final.jpeg", height = 3, width = 6)
```

```
filter(regeneration, control <18.18944184, length >300) $length[1]
```

```
## [1] 302.174
```

```
filter(regeneration, control_2 <15.33677786, length >300) $length[1]
```

```
## [1] 308.696
```

```
filter(regenerationrollingaverage, control_rollingwindow <18.18944184, length >300) $
length[1]
```

```
## [1] 302.174
```

```
filter(regenerationrollingaverage, Control_2_rollingwindow <15.33677786, length >300)
$length[1]
```

```
## [1] 310.87
```
